# Artificial Intelligence in Oculoplastics: A Survey-Based Study on Provider Perspective

**DOI:** 10.7759/cureus.81271

**Published:** 2025-03-27

**Authors:** Balazs Fazekas, Malik Moledina, Nehal Singhania, Katya Tambe

**Affiliations:** 1 Ophthalmology - Oculoplastics, Nottingham University Hospitals, Nottingham, GBR; 2 Ophthalmology, Queen Victoria Hospital, East Grinstead, GBR; 3 Ophthalmology, Nottingham University Hospitals, Nottingham, GBR

**Keywords:** artificial intelligence, oculoplastics, surgeons, survey, uk - united kingdom

## Abstract

Purpose

This survey aims to explore the attitudes of ophthalmologist oculoplastic surgeons in the United Kingdom (UK) towards specific advances in artificial intelligence (AI).

Methods

A web-based anonymised survey was distributed to oculoplastic surgeons in the UK between October 2023 and March 2024. The survey evaluated attitudes towards specific AI advances using a five-point scale. Questions were designed to ascertain understanding, application, as well as attitudes towards anticipated future developments, barriers and challenges of AI within the current oculoplastic clinical landscape.

Results

In total, 77 survey responses were analysed from oculoplastic surgeons practising around the UK. The majority of the responses were from oculoplastic consultants (64%, 44/69), with other responses from oculoplastic fellows (12%, 8/69), ophthalmology consultants (10%, 7/69), speciality training surgeons (6%, 4/69) and other ophthalmology clinicians (9%). The responses highlighted that there is a widespread level of subjective understanding of the topic of AI, a low level of current AI in clinical practice and an anticipation that AI will have a noticeable impact in the next one to five years (45%, 29/64) or five to 10 years (36%, 23/64) in the UK. Specific applications of applications of AI are also discussed in further detail.

Conclusions

Oculoplastic surgeons feel that AI will have an imminent role in this speciality, but there is a low level of self-rated knowledge and low current use of AI in clinical practice. Given the potential of this technology, further resources need to be invested into equipping Oculoplastic surgeons to feel more prepared for this technological revolution.

## Introduction

Artificial intelligence (AI) is the ability of a computer to mimic human intelligence and perform complex tasks such as learning, decision-making and event prognostication [1]. There has been a recent increase in research relating to AI in every sector of society, including in the medical field where the recent COVID-19 pandemic has necessitated more effective digitalisation of medical practice, in view of the imposed pandemic restrictions during this period [2].

Medical professionals have responded to this technology with a melee of anticipation and apprehension given the potential of AI to significantly impact clinical practice in both negative and positive ways [3-7]. Interestingly, whilst there are some parallels between the perceptions of different clinician groups towards AI, responses and attitudes towards it vary between both medical and surgical fields, as well as within individual subspecialties. It is likely that the ease and extent of integration of AI tools into clinical practice to attain good levels of performance will play a role in determining the adoption of AI tools by clinicians in each medical speciality [8]. Intriguingly, imaging-based disciplines such as ophthalmology, radiology, dermatology and oncology are expected to encounter a more significant impact in their speciality than less image-reliant disciplines [9].

The oculoplastics subspeciality is particularly well-suited for AI integration as it relies heavily on digital imaging and on objective metrics. Images from various modalities such as computed tomography (CT), magnetic resonance imaging (MRI) and high-resolution photography, provide the required data for machine learning (ML) to develop complex algorithms and models to execute tasks as required by clinicians. This is especially true where serial imaging is undertaken for monitoring and surveillance of pathology. Convolutional neural networks (CNNs), a type of deep learning model, are particularly effective at processing such multi-dimensional data and have begun to be used in various computational models already [10]. CNNs can be developed to process data quickly and accurately, but do require large datasets for training.

There have been a number of recent studies detailing specific applications of AI in the field of oculoplastics [11,12]. These highlight AI’s potential role in the diagnosis and monitoring of eyelid and orbital diseases such as orbital abscesses, thyroid-associated ophthalmopathy, and ptosis screening, as well as its potential for surgical planning. Despite the scope for impact, to our knowledge, there is limited data available on how oculoplastic surgeons in the UK perceive AI in general or their attitude towards the specific applications of AI in their speciality.

With this in mind, a survey was undertaken to assess the attitudes of oculoplastic surgeons towards AI in general, whilst also aiming to identify any areas for targeted research and development.

An understanding of these sentiments and identification of these topics will form a baseline against which future assessments can be compared. In addition, it may aid AI policy-makers and developers to focus their resources effectively in regard to this subspeciality.

We hypothesize that oculoplastic surgeons are positively disposed towards AI applications in their field of practice. Secondly, most surgeons would have limited experience and access to this technology. We feel that surgeons will perceive AI to have an important role as an adjunct to current clinical practices such as aiding in diagnostics and surgical planning.

## Materials and methods

An anonymised web-based survey was conducted using the SurveyMonkey (San Mateo, CA) tool, which is an all-in-one electronic software that helps to create professional surveys. The questions were developed with a focus group of three oculoplastic clinicians and were based on similar previous recent survey studies that assess the level of understanding on this topic [3-6,13]. (Please see Appendix A-D for the survey questionnaire used for the study.) The specific AI applications listed in the survey were chosen based on a review by Bao et al. (2022) that summarises the current impact of AI in oculoplastics [10]. The questions of the survey were made available on the British Oculoplastic Surgical Society (BOPSS) website and also sent out electronically to the membership. Oculoplastic surgeons are ophthalmologist doctors who specialise in both eyelid and facial plastic surgery relevant to the eyes. The survey was open between October 13, 2023, and March 2, 2024, for submitting answers and opinions. All participants who completed or partially completed the survey were included in the analysis. Survey questions that were left unanswered were excluded from the calculations.

The first section of the survey focused on general attitudes towards and experience with AI in clinical practice. The second part of the questionnaire required the participants to assign a level of importance on a five-point Likert scale to a series of present and future applications of AI within the oculoplastics speciality. A score of ‘1’ expressed that the participant felt that the AI application was ‘Least important’, and a score of ‘5’ an attitude that the application was ‘Most important’. Clinicians were asked to assign a level of importance to each oculoplastic area in which AI is likely to have an important role. The results of these responses were collated, and subsequently analyzed and are shown below. Statistical analysis of the results was carried out using IBM SPSS Statistics for Windows, version 20 (IBM Corp., Armonk, NY).

## Results

In total, 77 clinicians participated in the survey. The cohort comprised predominantly of oculoplastic consultants practising in the UK (64%, 44/69), with other responses from oculoplastic fellows (12%, 8/69), ophthalmology consultants (10%, 7/69), speciality training surgeons (6%, 4/69) and other ophthalmology clinicians (9%).

**Figure 1:**
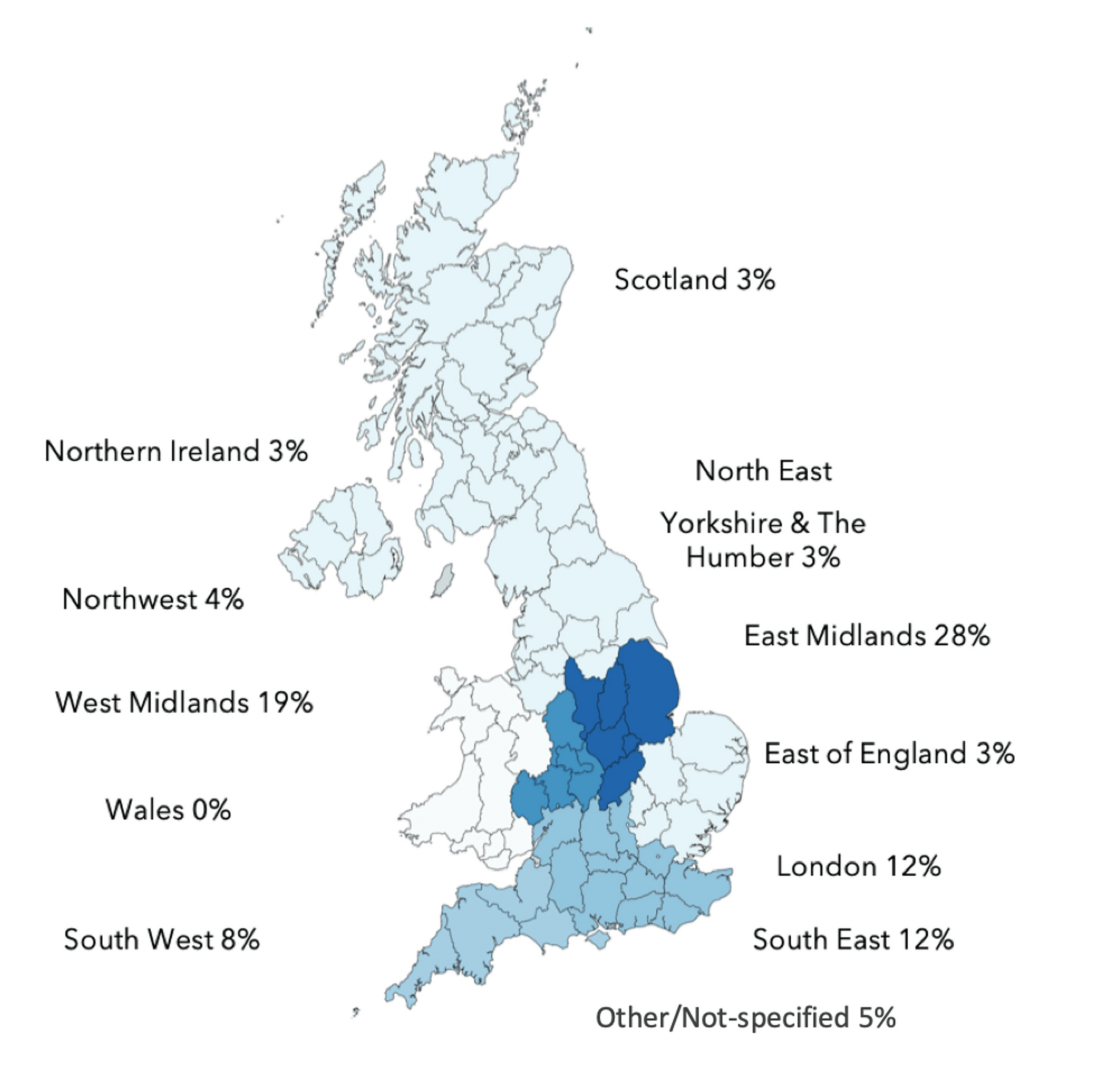
Regional representation of survey participation across the UK (n=75).

Most of the respondents were from the Midlands area, however, there was representation from around the UK (Figure 1). There was one response from a surgeon currently practising abroad.

The experience of participants within the oculoplastics speciality was quite evenly distributed varying between those with <5 years (29%, 20/70), five to 10 years (27%, 19/70), 10-15 years (11%, 8/70), 15-20 years (20%, 14/70) and those with over 20 years of experience (13% (9/70). The fallout rate for each question is demonstrated in the figures and varied between 0% (n=77/77) and 44% (n=34/77).

Section 1

The answers indicate that the majority of clinicians do not currently use AI in their clinical practice (91%, 61/67), but there were some respondents who reported using it on a monthly (6%, 4/67) and daily (3%, 2/67) basis. The current survey did not explore exactly how this is being achieved.

**Figure 2:**
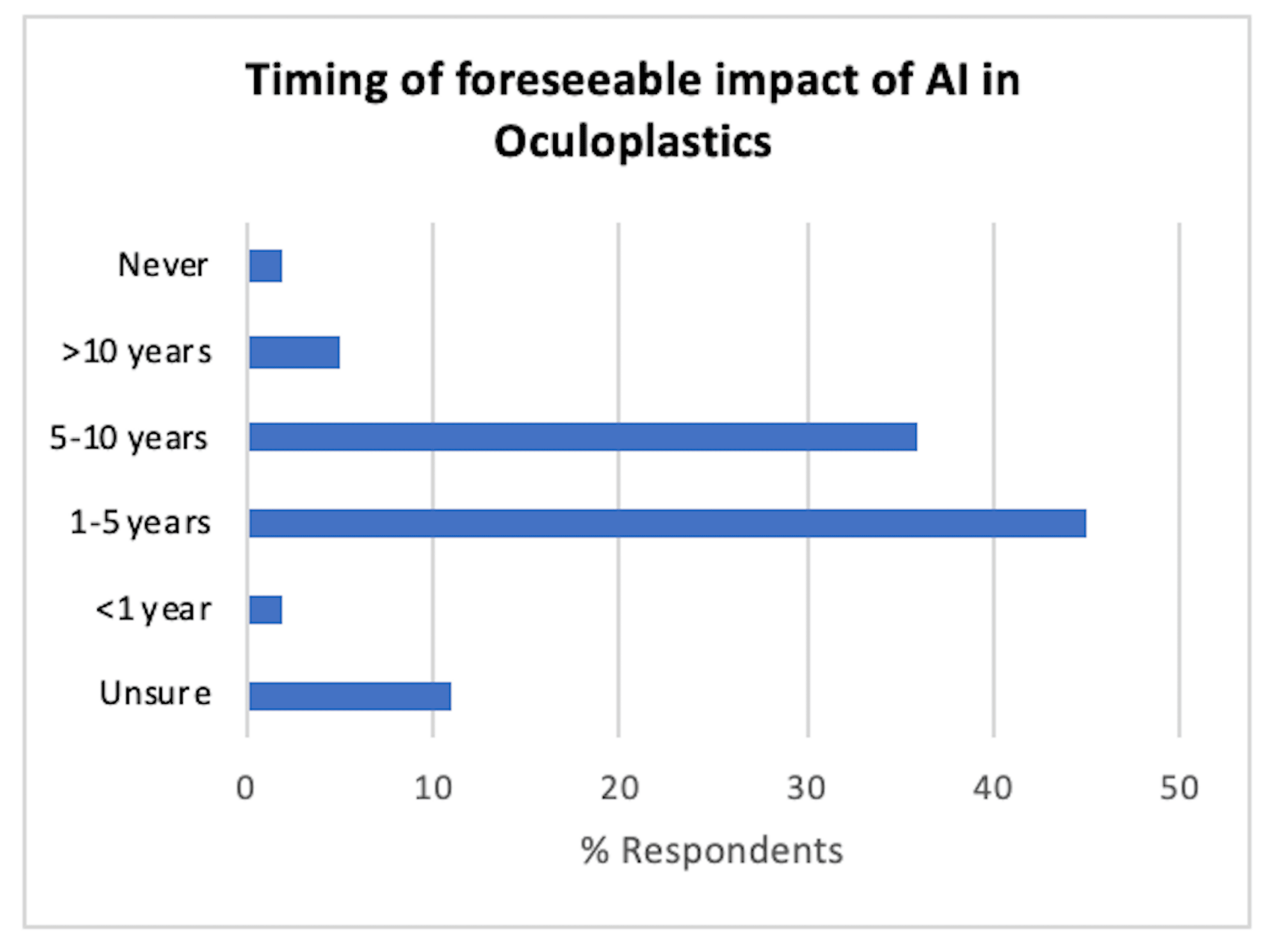
Timing of anticipated foreseeable impact of AI in oculoplastics (n=64). AI, artificial intelligence.

Most participants anticipate AI to have a noticeable impact in less than a decade, with specific timings listed in Figure 2. Specifically, participants anticipated to have a noticeable impact within one year (2%, 1/64), one to five years (45%, 29/64), five to 10 years (36%, 23/64), >10 years (5%, 3/64), never (2%, 1/64) and unsure (11%, 7/64).

Interestingly, the most common clinicians’ self-reported level of understanding of AI was ‘average’ (36%, 24/66)) and ‘below average’ (33%, 22/66). The remaining participants rated their level of understanding to be poor (15%, 10/66), above average (14%, 9/66) or excellent (2%, 1/66).

Section 2

**Figure 3:**
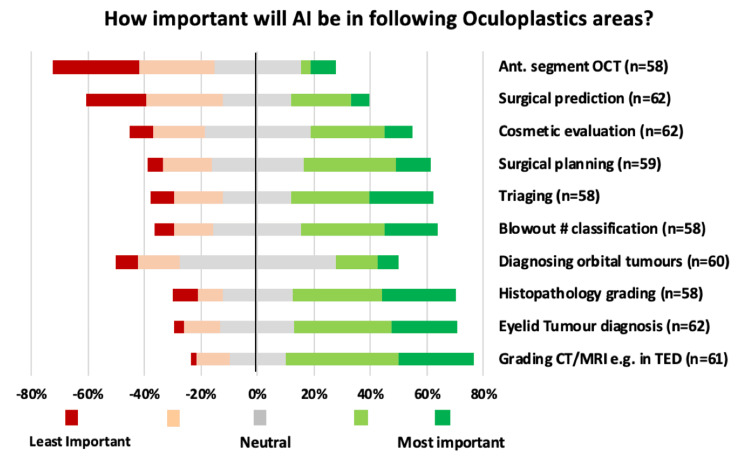
Summary of participants’ attitude towards individual AI applications. AI, artificial intelligence; OCT, optical coherence tomography; TED, thyroid eye disease.

A summary of the participants’ attitudes towards specific applications of AI in oculoplastics is shown in Figure 3. In order to aid interpretation, the rows have been re-ordered from ‘Least important’ at the top, to those deemed ‘most important’ at the bottom.

The areas of AI application with the highest average Likert-scale score was ‘Grading CT/MRI images in conditions such as thyroid eye disease’ (mean = 3.72 +/-1.03, n = 61 responses). The subsequent higher rated areas, in descending order, were ‘Eyelid tumour diagnosis via external photography’ (mean = 3.55 +/- 1.08, n = 62 responses), ‘Diagnosis and grading of tumour histopathology using AI’ (mean = 3.52 +/- 1.22, n = 58 responses), ‘Diagnosing, differentiating and monitoring orbital tumours’ (mean = 3.5 +/- 1.10, n = 60 responses) and ‘Classification of blow-out fractures using AI from orbital images’ (mean = 3.4 +/-1.15, n = 58 responses).

The area deemed to have the lowest importance, with the lowest average Likert-scale score, was ‘Using anterior segment OCT (optical coherence tomography) to assess tear meniscus and detect patients with nasolacrimal duct obstruction’ (mean = 2.26 +/-1.21, n = 58 responses). Furthermore, in ascending order, the topics with the lowest rated AI application scores were ‘Surgical prediction and post-operative evaluation of ptosis surgery outcomes using pre-operative external photographs’ (mean = 2.65 +/-1.22, n = 62 responses), ‘Objective evaluation of cosmetic surgery outcomes’ (mean = 2.65 +/-1.08, n = 62 responses), ‘Quantifying anatomical structures in surgical planning, e.g., orbital abscesses, orbital septal fat and navigation-guided surgeries’ (mean = 3.24 +/- 1.06), n = 59 responses) and ‘Using AI to screen triage letters from GPs or opticians’ (mean = 3.38+/-1.25, n = 58 responses).

**Figure 4:**
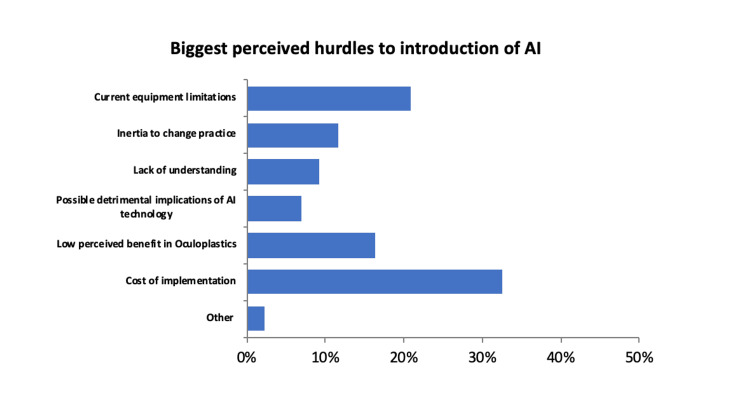
Summary of perceived biggest hurdle to the introduction of AI into clinical practice. AI, artificial intelligence.

The biggest perceived hurdles to the introduction of AI in oculoplastics were ‘cost of implementation’ (33%, 14/43), and ‘current equipment limitations’ (21%, 9/43) (Figure 4).

The other hurdles to the introduction of AI into clinical practice in descending order were low-perceived benefit in oculoplastics (16%, 7/43), inertia to change practice (12%, 5/43), lack of understanding (9%, 4/43), as well as ‘possible detrimental implications of AI technology’ (7%, 3/43).

**Figure 5:**
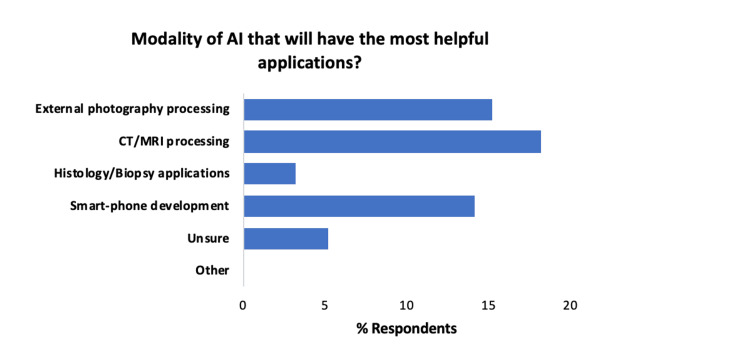
Summary of the modality that was deemed to be most likely to be impactful. AI, artificial intelligence.

The modality that was deemed to be most impactful as per the participants when introducing AI was its use in CT/MRI processing (33%, 18/55), external photography processing (27%, 15/55) and smart-phone developments (25%, 14/55) (Figure 5).

Some respondents felt unsure about which areas would be most helpful (9%, 5/55), and interestingly, histology/biopsy applications were only thought to be most helpful by a handful of clinicians (5%, 3/55).

Free text comments were invited at the end of the survey and raised valuable insights into the attitudes of oculoplastic surgeons towards this technology (Table 1).

**Table 1:** Free-text comments and related sentiment. AI, artificial intelligence; BOPSS, British Oculoplastic Surgical Society.

Comment	Idea
I am excited to explore ways that AI can improve quality of care and reduce administrative burden on clinicians	Supportive tool
I just wish I knew how to code/implement it! Having some national guidelines/support from BOPSS as things develop will be helpful in terms of business cases/safety profiles/training, etc.	Importance of understanding support welcome
Linking AI to current software is vital	Supplement current practice
Diagnostics	Role in some clinical aspects
I will be introducing AI to my practice initially for patient education in the next few months. Looking forward to have AI being used in the practice	Use imminent for some
I will have retired!	May not influence everyone’s practice

## Discussion

This survey was conducted in order to understand the perceptions of oculoplastic surgeons about AI. There is evidence that this subspeciality is particularly well-suited for AI integration as it uses extensive multi-modal digital imaging which could provide a large amount of training data for various artificial neural network (ANN) models and recent reviews have also suggested that AI could have an imminent role in this field [10-12,14]. This expectant sentiment was reflected in our study, in which the majority of our cohort anticipated a noticeable change brought in this speciality by AI within the next decade.

Despite these suggested imminent changes, our survey found that oculoplastic surgeons see a deficit in their self-reported knowledge and the majority do not yet implement AI applications in their current working environment, with only 6% reported using AI monthly and 3% daily. This finding is consistent with other studies assessing clinicians' attitudes towards AI. 

Pecqueux et al. (2022) studied the attitudes of 357 German surgeons and reported personal self-rated knowledge as average (41.6%) or rudimentary (37.3%) [6]. Sheetz et al. (2021) explored the attitudes of clinicians in four specialities in Australia with almost half of respondents (47.6%) rating their knowledge of AI as average relative to their peers; the use of AI in daily clinical practice in that study varied between 5.2-15.7% [3]. Oh et al. (2019) investigated the perceptions of doctors in Korea and noted that only 5.9% answered that they had good familiarity with AI [15].

In contrast, in the neurosurgical speciality, Staartjes et al (2020) reported a much higher AI usage with 38.5% of neurosurgeons reporting using ML in their clinical practice and 31.1% in their research [16].

The respondents of the cited surveys reiterate the need for education and training in AI via scientific meetings, seminars, the adoption of AI within the training curriculum, and closer collaboration with information technology teams. This opinion appeared in the present survey, as well. One of the current survey’s participants similarly invited more support and training from the BOPSS in the ‘free-text comments’ section of the survey. There was also a call for the development of national guidelines, which would be invaluable to help navigate through the practical challenges and ethical concerns.

The review by Bao et al. (2022) provides an overview of some of the possible avenues in which AI could be used in the oculoplastic speciality and this helped formulate the applications in the second section of the survey reported herein [10]. Grading and monitoring orbital diseases such as thyroid-associated orbitopathy using CT/MRI segmentation and AI received the highest Likert scale average score in our study.

Different ANNs have already been developed and trialled on datasets of both CT and MRI images to aid diagnosis and classify orbital diseases. Notable examples include using Inception V3 deep CNNs to accurately detect 92% of orbital burst fractures from CT scans by Li et al. (2020) [17]. 

In thyroid-associated orbitopathy (TAO), Song et al. (2021) identified TAO patients using CT scans using a three-dimensional (3D) Residual Neural Network (ResNet) model training with an area under the curve (AUC) of 0.919 with suggestions that this may provide an effective screening tool with further validation [18]. On a similar note, Lin et al. (2021) used a deep CNN based on parts from the Visual Geometry Group (VGG) and ResNet to identify and grade TAO patients using MRI images with good accuracy [19]. Furthermore, CNNs have been successfully trained to distinguish orbital cavernous venous malformations [20], determine if there is orbital tumour invasion [21] and distinguish between adnexal lymphoma from idiopathic orbital inflammation [22]. All of these advances have been published in the last four years and it is likely that this trend will continue.

Diagnosis of eyelid pathology using external photography AI systems was highly rated in our participant's Likert-scale rating score and has also been extensively studied recently. Notably, Li et al. (2022) developed a deep learning classification network (a faster region-based CNN) that uses tumour-centred heatmaps to automatically distinguish between malignant and benign eyelid tumours in photographic images (AUCs ranging from 0.899 - 0.955) [23]. This result is comparable to the evaluation carried out by a senior ophthalmologist and it might prove to be a valuable asset in carrying future screening from community referrals.

The results of the survey appear to be mostly in keeping with the initial hypothesis set out at the beginning of the article. Despite the outlined hurdles to the introduction of AI into clinical practice, there appears to be a perception that AI will have an important role imminently in this speciality potentially impacting both diagnostics and surgical planning.

This growing role of AI in oculoplastic surgery is further suggested by similar progress in the use of AI for surgeries involving the rest of the face. Stephanian et al. (2024) report improved diagnostic accuracy, predictive capabilities and aesthetic surgery treatment planning in facial aesthetic surgery using AI [24]. It is the author's opinion that similar AI models could be applied in these neighbouring branches of surgery in the future.

Limitations

Like other surveys of this kind, our study has notable limitations. Since our study aimed exclusively at oculoplastic surgeons, the sample size is relatively small for a large topic, which limits strong statistical conclusions. The BOPSS website reports over 200 oculoplastic surgeon members in the UK [25]. This study represents approximately one-third of the cohort of UK surgeons. The uptake ratio is similar to earlier similar surveys cited. In addition, there was a significant fall-out rate from the participants. This may be due to a myriad of factors including time constraints of oculoplastic surgeons or possibly since this topic is not imminently impacting their clinical practice. The self-reported low use of AI among surgeons may also affect the accuracy of their insight on this topic. Volunteer bias means that the results of our survey may not be representative of the views of all oculoplastic surgeons across the UK. In addition, the results of the survey may not be generalizable to all regions around the UK, as the majority of responses were from the Midlands area, limiting its transferability. Lastly, as the survey used predominantly structured responses (i.e., Likert scales), the scope of response options was limited and the survey findings may not give a fully comprehensive account of the views of the participants.

## Conclusions

This survey suggests that oculoplastic surgeons feel that AI will have an imminent role in this speciality, but there is a low level of self-rated knowledge and low current use of AI in clinical practice. Cost, equipment limitations and low-perceived benefits are the current reported hurdles to its widespread use. Nevertheless, many surgeons see applications of AI in CT/MRI processing, eyelid tumour diagnosis and histopathology grading amongst other uses in this speciality. Given the potential of this technology, further resources need to be invested into equipping oculoplastic surgeons through scientific meetings, the adoption of AI within the training curriculum, and closer collaboration with information technology teams in order to feel more prepared for this technology revolution.

## Figures and Tables

**Figure 6:**
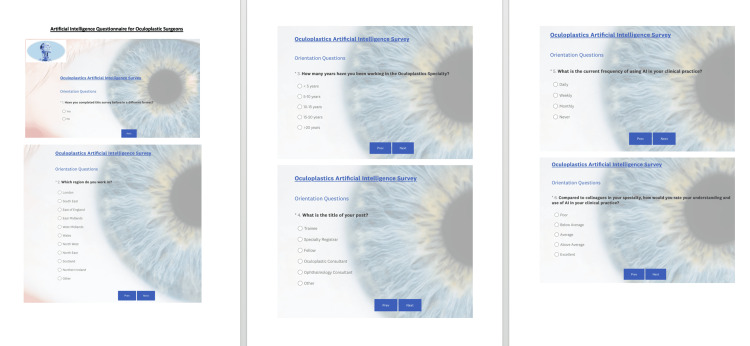
Original questionnaire on SurveyMonkey (questions 1-6).

**Figure 7:**
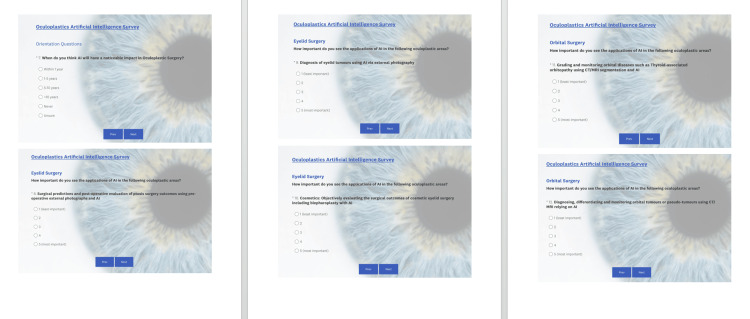
Original questionnaire on SurveyMonkey (questions 7-12).

**Figure 8:**
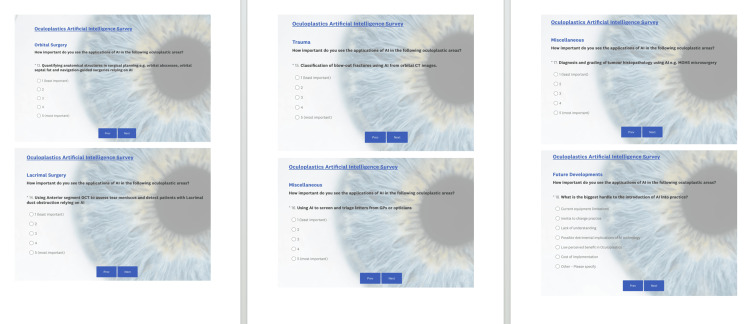
Original questionnaire on SurveyMonkey (questions 13-18).

**Figure 9:**
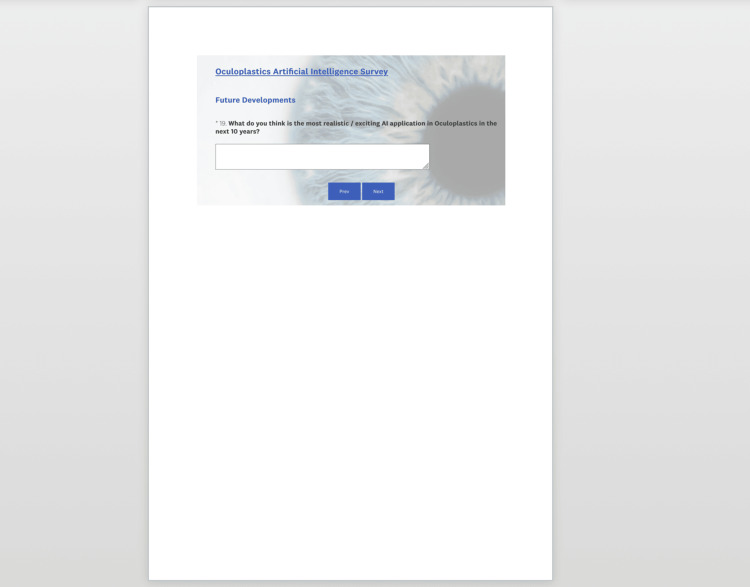
Original questionnaire on SurveyMonkey (question 19).

## Appendices

Appendix A 

Appendix B 

Appendix C 

Appendix D 
